# Investigation of Caucasian rheumatoid arthritis susceptibility loci in African patients with the same disease

**DOI:** 10.1186/ar4082

**Published:** 2012-11-03

**Authors:** Sebastien Viatte, Edward Flynn, Mark Lunt, Joanne Barnes, Madeleine Singwe-Ngandeu, Sylvette Bas, Anne Barton, Cem Gabay

**Affiliations:** 1 Arthritis Research UK Epidemiology Unit, Manchester Academic Health Science Centre, The University of Manchester, Oxford Road, Manchester, M13 9PT, UK; 2 Unit of Rheumatology, Department of Internal Medicine, School of Medicine, University of Yaoundé, Yaoundé, Cameroon; 3 Division of Rheumatology, University Hospitals of Geneva, Avenue Beau-Séjour 26, 1211 Geneva 14, Switzerland; 4 NIHR Manchester Musculoskeletal Biomedical research Unit, Central Manchester Foundation Trust, Manchester Academic Health Science Centre, Oxford Road, Manchester, M13 9PT, UK; 5 Department of Pathology & Immunology, University of Geneva School of Medicine, Rue Michel-Servet 1, 1211 Geneva 4, Switzerland

## Abstract

**Introduction:**

The largest genetic risk to develop rheumatoid arthritis (RA) arises from a group of alleles of the HLA *DRB1 *locus ('shared epitope', SE). Over 30 non-HLA single nucleotide polymorphisms (SNPs) predisposing to disease have been identified in Caucasians, but they have never been investigated in West/Central Africa. We previously reported a lower prevalence of the SE in RA patients in Cameroon compared to European patients and aimed in the present study to investigate the contribution of Caucasian non-HLA RA SNPs to disease susceptibility in Black Africans.

**Methods:**

RA cases and controls from Cameroon were genotyped for Caucasian RA susceptibility SNPs using Sequenom MassArray technology. Genotype data were also available for 5024 UK cases and 4281 UK controls and for 119 Yoruba individuals in Ibadan, Nigeria (YRI, HapMap). A Caucasian aggregate genetic-risk score (GRS) was calculated as the sum of the weighted risk-allele counts.

**Results:**

After genotyping quality control procedures were performed, data on 28 Caucasian non-HLA susceptibility SNPs were available in 43 Cameroonian RA cases and 44 controls. The minor allele frequencies (MAF) were tightly correlated between Cameroonian controls and YRI individuals (correlation coefficient 93.8%, p = 1.7E-13), and they were pooled together. There was no correlation between MAF of UK and African controls; 13 markers differed by more than 20%. The MAF for markers at *PTPN22, IL2RA, FCGR2A *and *IL2/IL21 *was below 2% in Africans. The GRS showed a strong association with RA in the UK. However, the GRS did not predict RA in Africans (OR = 0.71, 95% CI 0.29 - 1.74, p = 0.456). Random sampling from the UK cohort showed that this difference in association is unlikely to be explained by small sample size or chance, but is statistically significant with p<0.001.

**Conclusions:**

The MAFs of non-HLA Caucasian RA susceptibility SNPs are different between Caucasians and Africans, and several polymorphisms are barely detectable in West/Central Africa. The genetic risk of developing RA conferred by a set of 28 Caucasian susceptibility SNPs is significantly different between the UK and Africa with p<0.001. Taken together, these observations strengthen the hypothesis that the genetic architecture of RA susceptibility is different in different ethnic backgrounds.

## Introduction

Rheumatoid arthritis (RA) is a systemic and chronic inflammatory disease involving mainly the peripheral joints of hands and feet initially. RA is thought to arise from the combination of both genetic and environmental factors. The largest genetic risk arises from a group of alleles of the HLA-*DRB1 *gene, collectively referred to as the shared epitope (SE). Candidate-gene studies and genome-wide association studies (GWASs) have identified more than 30 non-HLA genetic loci to date [1]. The majority of single-nucleotide polymorphisms (SNPs) associated with RA susceptibility have been identified and validated in patients of European ancestry who were seropositive for either anti-citrullinated protein antibodies (ACPAs) or rheumatoid factor.

Several studies have shown that some validated RA risk alleles contribute to risk in other ethnic groups, in particular of Asian ancestry [2-4], and concluded that some RA susceptibility loci show population-specific associations but that others overlap between Asian and European populations.

Ethnic differences in allele frequencies of autoimmune disease-associated SNPs have been clearly shown [5], and a lack of association of a particular SNP in a population may be more likely to represent a lack of power due to low allele frequency than to represent a true negative effect at the individual level. The *PTPN22 *polymorphism rs2476601, a cardinal example, has an allele frequency of above 10% in healthy individuals of Northern Europe, 3% in Southern European countries, and 2% in Northern African and African-American populations but is absent in Asian populations [6]. However, most common SNPs associated with RA in persons of European ancestry confer risk of RA in African-Americans, whereas discordant genetic loci appear to be the minority [7,8]. Interestingly, the SE is associated with susceptibility to RA in African-Americans through European genetic admixture [9], and this is likely to be the case for several SNPs outside the HLA-region. African-Americans and Africans, therefore, have to be considered separately for epidemiologic and genetic studies.

Epidemiologic and genetic data on RA in African populations are scarce. RA genetics has been studied mainly in underpowered candidate gene association studies in Northern and Southern Africa, and there have been few reports in Tunisia (*PTPN22 *[10,11], *TNFAIP3 *[12], *STAT4 *[13], *IRF5 *[14], *DNASE1 *[15], *SUMO4 *[16], and *SLAMF1 *[17]), Morocco (HLA [18]), Egypt (*PADI4 *[19], *TRAF1/C5*, and *STAT4 *[20]), and South Africa (HLA [21], *IL-10 *[22], and *p53 *[23]). RA has uncommonly been reported in Black Africans in West and Middle Africa and its prevalence there is still unknown [24,25]. A few studies performed from the 1960s to the 1990s on the frequency of the disease suggested the rarity of RA in rural West Africa [26,27]. Recent reports from West and Middle Africa studied epidemiological, clinical, and serological profiles of RA patients in small series [25,28-31]. To the best of our knowledge, only two small genetic studies [29,32] have been performed so far in West/Middle Africa. A study performed in Senegal showed that the risk of developing RA was associated with HLA-DR10 but not HLA-DR4 [32]. We recently reported an important difference in the proportion of SE-positive patients in Cameroon compared with European patients, despite a similar proportion of anti-cyclic citrullinated peptide antibody (anti-CCP) positivity, suggesting that the contribution of other non-HLA genetic factors to disease susceptibility could also differ between Caucasians and Black Africans [29]. However, no genetic study of non-HLA markers of RA susceptibility has ever been performed in patients originating from ethnic groups of West or Middle Africa, although this region of Black Africa comprises over 350 million inhabitants.

Using a previously characterized cohort of Cameroonian patients with RA [29], we aimed to determine whether Caucasian non-HLA RA susceptibility loci are shared with Black African patients with RA, as has been shown for Black Americans [7]. Because the small size of the cohort prevented us from drawing any conclusions at the individual SNP level, we computed an aggregated genetic risk score (GRS) [33] to cumulatively test the association of Caucasian RA susceptibility SNPs as a whole with disease susceptibility in Cameroon.

## Materials and methods

### Patients and data collection

This study was carried out on 56 RA patients recruited from the outpatient Rheumatology Clinic of Yaoundé Central Hospital in Cameroon and on 50 healthy controls recruited among medical students and hospital workers in Yaoundé. Approval of the Cameroon National Ethical Committee was obtained prior to the study, and informed consent was obtained from all patients and controls included in this study. Detailed patient and control characteristics have been reported previously [29]. Publicly available genotypes from 119 unrelated Yoruba individuals in Ibadan, Nigeria (YRI) from the International HapMap Project, phases I+II+III, release #28 of August 2010 [34,35], were incorporated in the analysis. No phenotypic information (apart from gender) was collected with the YRI samples, and medical conditions of the donors are unknown. However, owing to the low prevalence of RA and other autoimmune conditions in Nigeria, the YRI samples have been considered to be non-RA samples and used as 'controls'. Publicly available allele frequencies calculated from 110 unrelated Luhya individuals in Webuye, Kenya (LWK) from the International HapMap Project (release #28) were retrieved by using SPSmart [36,37]. Nine thousand three hundred five individuals from the UK RA Genetics Consortium (UKRAG), comprising 5,024 RA cases and 4,281 controls, were available for analysis. Patients' and controls' characteristics and genotyping have been reported previously [38]. Anti-CCP status for Cameroonian and UK samples has been determined with the second-generation CCP (CCP2) assay.

### Single-nucleotide polymorphism selection

Caucasian confirmed RA susceptibility SNPs were selected from the large meta-analysis by Stahl and colleagues [1]. Apart from the HLA-DRB1*0401 tag (rs6910071), all SNPs, or good proxies (r^2 ^>0.9 using the 1,000 Genome reference panel for Caucasians - CEU), were available as part of a 67-SNP set used for high-throughput genotyping at our laboratory. This set of SNPs has been described in detail [39].

### Genotyping

Sixty-seven SNP markers were genotyped in Cameroonian samples by using Sequenom MassArray technology in accordance with the instructions of the manufacturer (Sequenom, San Diego, CA, USA). Patients with greater than 10% missing genotype data and SNPs with less than 90% genotyping success rate or in Hardy-Weinberg disequilibrium (*P* <0.001) were excluded, prior to analysis, as part of quality control procedures.

### Genetic risk score

A Caucasian GRS was computed according to the method of Karlson and colleagues [33]. Briefly, 28 Caucasian confirmed RA susceptibility SNPs with available genotype in the Caucasian (UKRAG) and African (Cameroon and YRI) populations were used. Genotypes were coded as 0, 1, or 2, corresponding to the number of risk alleles carried by an individual. Missing genotypes in UKRAG cases, controls, African cases, or controls were replaced by twice the risk allele frequency of UKRAG cases, controls, African cases, or controls with available genotypes, respectively. The natural logarithms of the odds ratios (ORs) taken from the meta-analysis by Stahl and colleagues (Table 2 of [1]) were used to weight the risk allele count and compute the GRS. For the newly identified susceptibility markers by Stahl and colleagues (Table 3 of [1]), the ORs from the replication analysis were used in order to avoid the 'winner's curse' effect.

### Statistical analysis

All quality control steps were performed by using the PLINK software package [40]. Subsequent statistical analysis was performed with Stata version 10.1 (Stata Statistical Software: Release 10; StataCorp LP, College Station, TX, USA). Correlation calculations of minor allele frequencies (MAFs) between different cohorts/populations were performed by using linear regression. Association analyses between disease susceptibility and GRS were performed with logistic regression.

## Results

### Cohort characteristics and genotyping results

Cameroonian RA cases and controls collected previously [29] were genotyped for a set of 67 susceptibility SNPs. Basic cohort characteristics of the 43 Cameroonian patients and 44 controls available for analysis after genotyping and quality control procedures do not differ from the characteristics of the entire cohort as presented previously [29] and are compared in Table 1 with basic characteristics of UK and YRI individuals. Only independent SNPs from the meta-analysis by Stahl and colleagues were considered for our analysis, which consisted of a set of 32 non-HLA susceptibility SNPs. rs934734 (*SPRED2*) and rs540386 (*RAG1/TRAF6*) completely failed genotyping. rs6859219 (*ANKRD55/IL6ST*) and rs10488631 (*IRF5*) genotypes or proxies were not available in UKRAG. Therefore, 28 independent confirmed Caucasian RA non-HLA susceptibility markers (Table 2) had available genotypes for analysis in the three datasets (Cameroon, UKRAG, and YRI). In addition, genotypes for rs6910071, the HLA-DRB1*0401 tag [1], were available for YRI individuals, and four-digit HLA typing was available for the Cameroonian cohort and UKRAG.

**Table 1:** Summary of cohort characteristics

	Cohort				
**Characteristics**	**UKRAG**		**Cameroon**		**YRI HapMap^a^**

Health status	1987 ACR RA^b^	Controls	1987 ACR RA^b^	Controls	Controls^c^

Number	5,024	4,281	43	44	119

Females, percentage	72	66	95	68	50

Age (IQR), years^d^	50 (39-61)	-	44 (29-57)^e^	-	-

CCP-positive, percentage^f^	67	-	84	-	-

Erosive patients, percentage	65	-	51	-	-

^a^Unrelated individuals from the Yoruba in Ibadan, Nigeria (YRI) population, from the International HapMap Project. ^b^Satisfying the 1987 American College of Rheumatology classification criteria for rheumatoid arthritis (RA). ^c^Since no phenotypic information was collected by the HapMap Consortium, medical conditions of the YRI individuals are unknown; based on the assumed low prevalence of RA in Nigeria, 'controls' means that YRI individuals are unlikely to have RA and were considered to be controls. ^d^Median patient age at disease onset, with interquartile range (IQR). ^e^The median age (IQR) at inclusion is 52 (37-61) and therefore does not differ from the age reported for the 56 patients in the original paper [29]. ^f^Positive for anti-cyclic citrullinated peptide antibody (anti-CCP). For samples tested on several occasions over time, anti-CCP status was defined as positive if at least one of the tests was positive ('ever positivity'). Disease duration at the time of the last x-ray differs between patients and cohorts. The anti-CCP status was available for only 2,842 United Kingdom Rheumatoid Arthritis Genetics Consortium (UKRAG) patients, the rheumatoid factor status was available for 4,709 patients, and x-ray data were available for 2,851 patients, whereas there was almost no missing information in the Cameroonian cohort.

**Table 2:** Single-nucleotide polymorphisms used to compute the genetic risk score

Chromosome	Locus name	SNP markers
1	*CD2/CD58*	rs11586238

1	*FCGR2A*	rs12746613

1	*MMEL1/TNFRSF14*	*rs10910099*

1	*PTPN22*	rs2476601

1	*PTPRC*	rs10919563

2	*AFF3*	rs10865035

2	*CD28*	rs1980422

2	*CTLA4*	rs3087243

2	*REL*	rs13031237

2	*STAT4*	rs7574865

3	*DNASE1L3/PXK*	rs13315591

4	*IL2/IL21*	rs6822844

4	*RBPJ*	rs874040

5	*GIN1/C5orf30*	rs26232

6	*CCR6*	rs3093023

6	HLA-*DRB1 *0401 tag	rs6910071

6	*PRDM1*	rs548234

6	*TAGAP*	rs394581

6	*TNFAIP3 *locus 1	rs6920220

6	*TNFAIP3 *locus 2	*rs13192841*

6	*TNFAIP3 *locus 3	rs5029937

8	*BLK*	rs2736340

9	*CCL21*	rs2812378

9	*TRAF1/C5*	*rs10760130*

10	*IL2RA*	rs2104286

10	*PRKCQ*	rs4750316

12	*KIF5A/PIP4K2C*	rs1678542

20	*CD40*	rs4810485

22	*IL2RB*	*rs3218258*

The 28 non-HLA independent rheumatoid arthritis confirmed susceptibility single-nucleotide polymorphisms (SNPs) plus the HLA-DRB1*0401 tag from the meta-analysis by Stahl and colleagues [1] or proxies (in italics in the third colomn) used in the present study are shown here.

### Minor allele frequencies of Caucasian rheumatoid arthritis susceptibility single-nucleotide polymorphisms differ importantly between West/Central Africans and Caucasians

**Figure 1:**
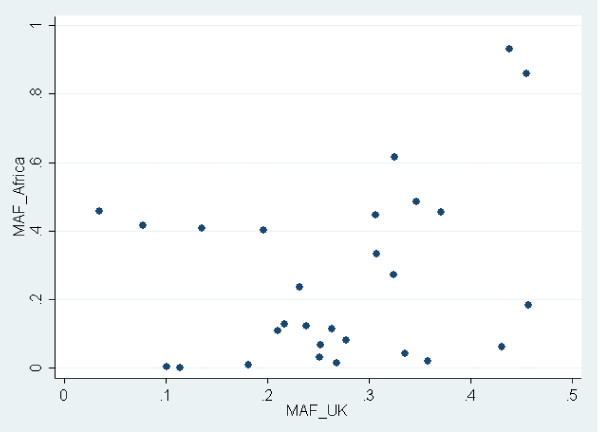
**Lack of correlation between minor allele frequencies (MAFs) of non-HLA rheumatoid arthritis susceptibility single-nucleotide polymorphisms in Caucasians (UK) and Africans**. The MAF of each polymorphism presented in Table 2 in the UK (MAF_UK) is compared with the frequency of the same allele in Africa (MAF_Africa). 'Minor' refers to a frequency of lower than 50% in the UK. Single-nucleotide polymorphisms with an 'MAF_Africa' of greater than 50% are minor alleles in the UK but major alleles in Africa.

Nigeria and Cameroon are neighboring countries from Central/West Africa. Although no MAF filtering was applied and the number of Cameroonian controls was very low, the MAFs of the 28 Caucasian RA susceptibility SNPs were very tightly correlated between Cameroonian controls and YRI individuals (correlation coefficient = 93.8%, *P *= 1.7 × 10^-13^). Therefore, YRI and Cameroonian controls were pooled together for further analysis and referred to as African samples, totaling 206 individuals (43 cases and 163 controls). Interestingly, the MAF of Caucasian RA susceptibility SNPs showed no correlation at all between West/Central African controls and UK controls (correlation coefficient = 30.4%, *P *= 0.115) (Figure 1) and differed importantly for most of the markers considered here: out of 28 SNPs, only 6 differ by less than 10% and 13 differ by more than 20%. rs10760130 (*TRAF1/C5*) shows the strongest difference, with an allele frequency of 44%, in the UK for the G allele (minor and risk allele), whereas the G allele is the major allele, with a frequency of 93%, in Africa. Although the MAF is between 10% and 27% for rs12746613 (*FCGR2A*), rs2476601 (*PTPN22*), rs6822844 (*IL2/IL21*), and rs2104286 (*IL2RA*) in the UK controls, the MAF for those polymorphisms is below 2% in African controls. By contrast, rs5029937 (*TNFAIP3*) and rs13315591 (*DNASE1L3/PXK*), which display low MAFs in the UK (3.5% and 7.8%, respectively), show a frequency of above 40% in Africa.

### Caucasian non-HLA RA susceptibility SNPs, considered in aggregate, do not confer disease risk in Africa

**Figure 2:**
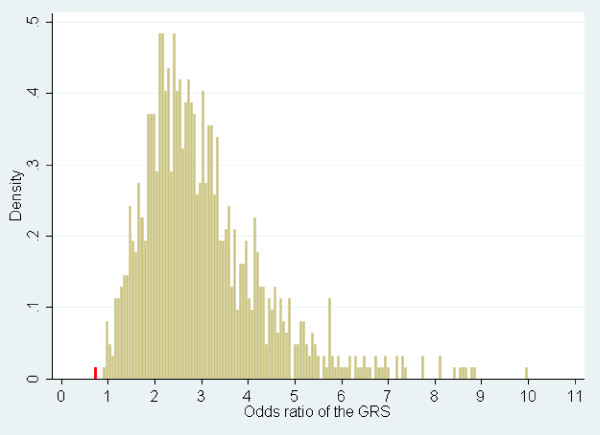
**The genetic risk of developing rheumatoid arthritis (RA) conferred by a set of 28 Caucasian non-HLA susceptibility single-nucleotide polymorphisms is significantly different in the UK and in Africa**. One thousand random samples of 43 RA cases and 163 controls (corresponding to the sample size of the African dataset) were generated out of a UK dataset comprising 5,024 RA cases and 4,281 controls. The distribution of odds ratios for association between the Caucasian genetic risk score (GRS) and disease susceptibility is shown in yellow for Caucasian random samples and in red for Africans.

The low number of African RA cases prevented an association study at the individual SNP level. To test the cumulative association of 28 Caucasian non-HLA confirmed RA susceptibility SNPs, we computed a weighted Caucasian GRS, a widely used strategy in complex disease genetics to summarize the effect of multiple markers within a single variable [33,41-44]. The Caucasian GRS showed a strong and highly significant association with RA in UKRAG (OR = 2.87, 95% CI 2.60 to 3.17, *P *= 2.7 × 10^-96^) when all UKRAG cases and controls were incorporated in the analysis. To investigate the association of the GRS in small samples, 1,000 random samples of 43 UKRAG cases and 163 UKRAG controls were tested and the distribution of the ORs was plotted (Figure 2). The 1,000 ORs ranged from 0.93 to 10.00. The Caucasian GRS was computed for the African samples but did not predict RA in this population (OR = 0.71, 95% CI 0.29 to 1.74, *P *= 0.456). By contrast, the OR was smaller than the smallest of the 1,000 ORs obtained in UK samples of the same size (red bar in Figure 2), a situation likely to occur by chance in less than 1 in 1,000 cases. The cumulative association of the 28 Caucasian non-HLA RA susceptibility SNPs is therefore significantly different between the UK and Africa (*P* <0.001).

Although the effects of HLA-*DRB1 *SE alleles have been previously studied in the Cameroonian cohort, we computed a GRS containing one more parameter: the HLA-DRB1*0401 (hereafter, GRS_0401). rs6910071 was used as a surrogate marker in YRI. GRS_0401 was strongly associated with RA in URKAG (OR = 3.43, 95% CI 3.17 to 3.71, *P *= 8.7 × 10^-206^), the ORs of 1,000 random samples ranged from 1.29 to 10.35, and the gap with the OR in Africans (OR = 0.51, 95% CI 0.22 to 1.16, *P *= 0.107) was even larger.

## Discussion

The prevalence of HLA-*DRB1 *SE alleles is highly variable across populations. Across Europe, for example, the prevalence of HLA-DRB1*0401 has been shown to vary from 4.3% in Spain to 24% in Sweden [45]. A higher prevalence of SE alleles in a population seems to correlate with a higher prevalence of RA [46]: the frequency of the SE is 59% in the Cree and Ojibway, a North American Native people in central Canada who have a higher prevalence of RA than Caucasians living in the same area. Ethnic variations in both the frequency and types of SE-carrying HLA-*DRB1 *alleles have been reported across different populations [47], but very few studies have been performed in Africa. Previously, we reported a lower prevalence of SE alleles in Cameroon and reviewed the scarce literature on studies performed in Black Africans [29]. In the present study, we investigated the contribution of non-HLA genetic markers to RA susceptibility in Cameroon. In a first step, we compared the MAFs of those polymorphisms between different populations. In a second step, we tested the association of confirmed Caucasian non-HLA RA susceptibility loci 'in aggregate' with disease susceptibility since the number of Cameroonian patients with RA was very low. No study of non-HLA genetic markers of RA susceptibility has ever been conducted before in Black Africans from Central/West Africa.

We pooled YRI individuals and Cameroonian controls on the basis of the observation that MAFs are very similar between those two datasets. Although samples have been collected from two neighboring countries, population stratification issues are possible. The low number of SNPs and samples tested here prevented us from addressing this possibility directly. There are more than 2,000 distinct language groups in Africa; ethnic origin, language, geographical location, and genetic differences correlate, so that population structure and genetic diversity are much larger on the African continent than anywhere else in the world [48,49]. Tishkoff and colleagues [49] studied 113 geographically diverse African populations, including 37 Cameroonian (including Bamoun, Lemande, and Bulu) and 9 Nigerian (including Yoruba) populations. Phylogenetic and population structure analyses showed that most of the Cameroonian populations are closely related genetically and that the genetic distance between them and Yoruba is small. Most Nigerian and Cameroonian populations belong to the West/Central Africa population cluster. However, the East Africa cluster - containing, for example, the Luhya population in Webuye, Kenya (LWK) - is genetically more distant. As an indirect way to estimate the putative impact of within-population structure on our results, we compared the MAFs of non-HLA RA susceptibility loci between Cameroonian controls and LWK individuals (MAFs of 24 SNPs out of 28 studied here were publically available for LWK; see Materials and methods). MAFs of Cameroonian controls and LWK individuals were as tightly correlated (correlation coefficient = 95.1%, *P *= 1.2 × 10^-12^) as were MAFs between Cameroonian controls and YRI individuals (correlation coefficient = 93.8%, *P *= 1.7 × 10^-13^). However, there was no correlation between MAFs of LWK and UK controls (correlation coefficient = 22.6%, *P *= 0.29). These findings are compatible with the out-of-Africa hypothesis of human origins and the associated population bottlenecks, which apparently resulted in the alteration of the MAFs studied here. Given the similarity in MAFs across African populations that are more distantly related (West and East Africa), within-population admixture in our case control study of Yoruba/Cameroonian individuals is unlikely to confound the findings. Moreover, population stratification issues are less likely to affect conclusions based on an aggregate GRS than conclusions at the individual SNP level.

Although the sample size is small, there is no doubt that the risk allele frequency of Caucasian RA susceptibility loci is very different in the African samples available. The vast majority of Caucasian susceptibility loci are common genetic variants for reasons related to study design and power issues. Polymorphisms with an allele frequency close to 50% should be detected even in small samples; however, several were barely detectable in the African control samples.

As expected, the Caucasian GRS showed a strong and significant association in the large UK cohort studied here. Even when 1,000 random samples of as few as 43 UK RA cases and 163 UK controls (size of the African sample) were tested for the association of the GRS with RA, the vast majority of them showed an OR of larger than one (997 of 1,000) or a *P *value of smaller than 0.05 (763 of 1,000). The OR in the African sample was much smaller than the smallest OR in the UK, a situation that is extremely unlikely to be explained by small sample size (*P *<0.001). The effect size ratio - that is, OR_UK_/OR_Africa _- is 4.04 (95% CI 1.6 to 10.0). Such a striking difference using a small number of Cameroonian RA samples highlights an important difference in the genetic architecture of RA susceptibility between the UK (or Caucasians) and Cameroon (West/Central Africa).

Several hypotheses can be postulated to explain the effect size ratio. First of all, the currently known Caucasian RA susceptibility loci might constitute a minority of the total RA susceptibility loci and might not be shared with African patients. Indeed, many more susceptibility variants, possibly thousands of SNPs, could contribute to the susceptibility to RA [50]. Secondly, even if those markers are shared, their effect size might be different between Caucasians and Africans. This would explain on its own the difference observed since the Caucasian GRS has been computed by using the Caucasian effect sizes. Thirdly, the confirmed RA susceptibility loci from the meta-analysis by Stahl and colleagues [1] might not be the causal SNPs in Caucasians but SNPs in linkage disequilibrium (LD) with the causal SNP. Fine-mapping experiments for most of those loci have not been performed yet. Therefore, the causal SNP, even if shared between the two populations, might be in tight LD with the Stahl SNPs in Caucasians but not in Africans. Under this hypothesis, an effect size ratio of 4.04 indicates that many of the Stahl SNPs might not be causal. This difference in the genetic architecture of the human genome between Africans and Caucasians can be further illustrated at the *MMEL1/TNFRSF14 *locus; rs10910099 was used as a proxy for the Stahl SNP rs3890745. The LD (r^2^) between these two markers is 0.926 when the 1,000 Genome CEU (Caucasian) is used as a reference panel but is 0.662 when the 1,000 Genome YRI panel is used. It is not known which one of the two SNPs, if either, is the causal.

Including the HLA-DRB1*0401 allele in the computation of the GRS further increased the gap between the association pattern in the UK versus Africa, consistent with previously published observations on the low allele frequency of the HLA-DRB1*0401 allele in African patients with RA [29,32]. Therefore, the genetics of RA differs significantly between the UK and Cameroon in terms of allele frequencies of currently known HLA and non-HLA markers and their aggregate effect on disease susceptibility. Further studies are required in larger samples of African RA patients of different origins to investigate whether this statement can be generalized for Caucasians and Black Africans. Owing to the difference in the LD structure between Caucasians and African populations, well-powered GWASs of patients with RA will greatly increase our understanding of the contribution of genetic factors to disease susceptibility and allow deeper fine-mapping experiments.

## Conclusions

An aggregate GRS based on the independent and confirmed non-HLA Caucasian RA susceptibility SNPs predicts RA in the UK but not in West/Central Africa. The difference in association between the GRS and disease susceptibility is so large that it can be significantly detected even when only 43 Cameroonian RA samples are included in the analysis. This observation strengthens the hypothesis that the genetic architecture of RA susceptibility is different in different ethnic backgrounds and suggests that currently known RA susceptibility SNPs are different or have different effect sizes between Caucasians and Africans. Well-powered GWASs of African patients with RA are required to identify susceptibility loci specific to ethnic groups and to contribute to the fine mapping of currently known loci.

## Abbreviations

anti-CCP: anti-cyclic citrullinated peptide antibody; CEU: Utah residents with ancestry from northern and western Europe; CI: confidence interval; GRS: genetic risk score; GWAS: genome-wide association study; LD: linkage disequilibrium; LWK: Luhya individuals in Webuye: Kenya; MAF: minor allele frequency; OR: odds ratio; RA: rheumatoid arthritis; SE: shared epitope; SNP: single-nucleotide polymorphism; UKRAG: United Kingdom Rheumatoid Arthritis Genetics Consortium; YRI: Yoruba in Ibadan: Nigeria.

## Competing interests

The authors declare that they have no competing interests.

## Authors' contributions

SV performed the statistical analysis and drafted the manuscript. EF performed the genotyping and helped to process collected clinical samples. ML participated in the statistical analysis. JB and SB helped to process collected clinical samples. CG is one of the founders and principal investigators on the Cameroonian cohort and helped to conceive the study and participated in its design and coordination. MS-N is one of the founders and principal investigators on the Cameroonian cohort and collected phenotypic information and clinical samples. AB helped to conceive the study and participated in its design and coordination. All authors read and approved the final manuscript.
